# The Development of Environmentally Sustainable Poly(vinyl chloride) Composite from Waste Non-Metallic Printed Circuit Board with Interfacial Agents

**DOI:** 10.3390/polym15132938

**Published:** 2023-07-04

**Authors:** Aung Kyaw Moe, Jirasuta Chungprempree, Jitima Preechawong, Pornsri Sapsrithong, Manit Nithitanakul

**Affiliations:** 1 The Petroleum and Petrochemical College, Chulalongkorn University, Bangkok 10330, Thailand; aungkyawmoe.cu.th@gmail.com (A.K.M.); jirasuta.chung@gmail.com (J.C.); jitima.pre@gmail.com (J.P.); 2 Center of Excellence on Petrochemical and Materials Technology, Bangkok 10330, Thailand; 3 Department of Mechanical Engineering Technology, College of Industrial Technology, King Mongkut’s University of Technology North Bangkok, Bangkok 10800, Thailand; pornsri.s@cit.kmutnb.ac.th

**Keywords:** waste non-metallic printed circuit board, interfacial agents, poly(vinyl chloride)

## Abstract

The recycling of non-metallic printed circuit boards (NMPCB) as a filler in poly(vinyl chloride) (PVC) composite would help to encourage the use of waste NMPCB, thus, reducing some environmental concerns with regard to e-waste. The objective of this study was to comprehensively evaluate the effect of different interfacial agents, namely polypropylene grafted maleic anhydride (PP-g-MAH) and ϒ-aminopropyltriethoxy silane (ATPS) on the morphology and properties of PVC/NMPCB composites. A PVC/NMPCB composite was prepared by melt compounding with varying amounts of NMPCB ranging between 10, 20 and 30 wt.%. Fourier transform infrared spectroscopy–attenuated total reflectance (FTIR–ATR) analysis revealed the interactions between PVC and NMPCB when using both PP-g-MAH and ATPS interfacial agent. The properties and morphology of PVC/NMPCB composites were significantly dependent on the interfacial agent treated on the NMPCB surface. The phase morphology and mechanical properties of PVC/NMPCB composites (30 wt.% of NMPCB) were improved and the result also indicated that the higher compatibility of composites with ATPS as an interfacial agent led to our obtaining the maximum Young’s modulus of 484 MPa. The dynamic mechanical analysis revealed the interaction at the interface, with the T_g_ shifting to a lower temperature in the presence of PP-g-MAH and strong interfacial adhesion noted with the improved T_g_ in the presence of the ATPS interfacial agent. Further evidence of the improved interaction was observed with the increment in density in the presence of ATPS when compared with PP-g-MAH in PVC/NMPCB composite. Hence, of the two interfacial agents, ATPS showed itself to be more effective when employed as an interfacial agent for NMPCB in PVC composite for industry.

## 1. Introduction

It is estimated that waste from electrical and electronic equipment (WEEE) or e-waste in the global waste stream is increasingly generated due to the short life span of equipment, continuous development of technology and rapid production, together with the fast consumption rate of electronic equipment. Printed circuit board (PCB), with metal and non-metal components (such as thermoset resin, glass fibers, etc.), is the most fundamental part in electrical and electronic products. Therefore, the generation of waste PCB is rising rapidly. The inappropriate management of end-of-life WEEE results in the depletion of materials and pollution of the environment. Until now, the recycling (materials’ recovering) of printed circuit board (PCB) has mainly focusing on the recovery of valuable metals (Au, Ag, Pt and Pd).

However, the recycling of the non-metallic fraction (NMF) of waste PCB was neglected and carried out improperly, i.e., using landfill and incineration, releasing toxic substances into soil, water and air that negatively impacted on the environment. Therefore, the growth of environmental regulations at global and regional level, driven by public concern, has led to more eco-friendly and effective recycling of WEEE or e-waste [1]. Various researchers have searched for proper waste management techniques to mitigate the risk to human health and of environmental degradation [2,3,4,5]. The non-metallic fraction (NMF) of waste PCB is made from polymers obtained from non-renewable petroleum resources, resulting in the energy crisis and resource depletion. Consequently, three types of recycling methods for nonmetallic parts of PBC waste have been proposed; these are chemical recycling (pyrolysis, depolymerization), thermal recycling and mechanical recycling. It has been reported that the pyrolysis process (thermal treatment) of PCB waste is a promising method for obtaining alternative fuels, along with the recovery of glass fiber, carbon and metallic fractions. C. Quan et al. reported that the pyrolysis oil from PCB contained high molecular weight organic species bromophenols, bisphenol A, p-isopropenyl phenol, phenol, etc. [4]. Furthermore, C. Ma et al. studied the characterization of pyrolysis of PCB waste with a view to the recovery of valuable metals from pyrolysis oil containing phenol derivatives that have potential for the synthesis of phenolic resin [6]. R.A. Alenezi et al. demonstrated that the products from the pyrolysis of PCB waste could be used for energy and the non-combustible material as a filler in the cement and brick industry [7]. F.R. Xua investigated the recovery of valuable metals and monomers or some useful chemicals from waste PCB using the supercritical methanol method [8]. To better understand the reaction rate and minimize side reactions, the various pyrolysis methods still require further improvements. Additionally, researchers are concentrating on the mechanical recycling of PCBs using a more straightforward and efficient process [9].

Currently, many researchers are focusing on the reutilization of NMPCB from waste printed circuit board (PCB) as a filler in polymer composites as a feasible strategy with low cost and high usage of NMPCB. The incorporation of NMPCB powder into polymer matrixes could produce outstanding properties such as improved thermal stability [10], flame retardancy and smoke suppression [11]. An example was the utilization of PCB waste by-product, following the recovery of the metallic fraction using the bioleaching process, to create polyvinyl alcohol membranes for the treatment of waste water [12] and for making supercapacitors [13].

Nevertheless, the incorporation of waste NMPCB into polymer matrixes showed poor interfacial adhesion due to poor compatibility between the NMPCB and the polymer matrixes as a result of different polarity between the matrix and filler. This poor compatibility adversely affects the adhesion between the NMPCB powder and the polymer matrix [14,15], resulting in poor mechanical properties. Therefore, development of a method which could improve the mechanical properties of the composites is one of the pressing tasks for the researchers. It could also be seen that the utilization of compatibilizer in NMPCB/polymer and the surface modification of NMPCB were effective methods to improve the properties of the NMPCB/polymer composite [16]. Consequently, compatibilizers were employed in the development of recycled NMPCB from waste PCB/recycled polymer composites such as recycled polypropylene [17,18,19,20], recycled polyethylene [21] and recycled ABS [22].

Previous studies by R.M. Grigorescu et al. illustrated the incorporation of NMPCB from 0 wt.% to 30 wt.% in recycled PVC (wire grade) using the solvent casting method [23]. Rajesha K Das et al. showed that poor mechanical properties were obtained in NMPCB/recycled PVC (wire grade)—a result of poor compatibility [24]. X. Wang et al. demonstrated the incorporation of glass fiber from paper based NMPCB in PVC [25] and they found that the glass fiber diameter and contents governed the tensile strength and bending strength of the resulting PVC composite. Consequently, compatibility between NMPCB and polymer matrixes was found to be related to NMPCB from the different sources of WEEE and the type of PVC matrix employed since the formulation of PVC from different additives was employed for the desired mechanical properties of the different products.

Our previous study reveals that the NMPCB was successfully modified with 1 wt.% of GPTMS, which promoted dispersion and interfacial adhesion in the PVC matrix, resulting in better tensile and thermal properties of the PVC/NMPCB composite [10]. The objectives of this work were to reduce environmental impact, lessen resource depletion and promote a more effective and sustainable recycling system for exploiting NMPCB using the novel recycling method of extracting NMPCB from waste PCB in PVC composite production. This includes the incorporation of NMPCB from waste PCB (glass fiber base, namely FR-4 type) together with the application of different interfacial agents in PVC composite. This research utilized different interfacial agents, with different chemical structures, which are commonly used in industry and are relatively inexpensive, using different methods of surface modification and addition of compatibilizers. The study examined and compared the beneficial effect of the methods on the mechanical and viscoelastic properties of the resulting PVC composite. The interactions between the treated NMPCB and PVC matrixes using ATR-FTIR analysis were also investigated. Furthermore, the effect of different interfacial agents (PP-g-MAH and ATPS) on Young’s modulus and the tensile strength of waste PVC/NMPCB composites was investigated. Scanning electron microscopy was also employed to study the dispersion of NMPCB filler in the polymer matrix. The extent of interaction adhesion in two different interfacial agents on NMPCB in PVC matrix was evaluated using dynamic mechanical analysis (DMA).

## 2. Materials and Methods

### 2.1. Materials

**Figure 1:**
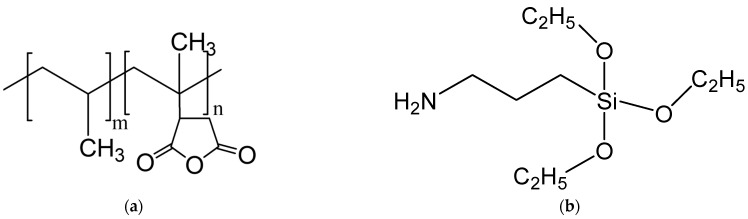
Chemical structure of interfacial agents (**a**) polypropylene graft maleic anhydride (PP-g-MAH) and (**b**) ϒ-aminopropyltriethoxysilane (ATPS).

Poly(vinyl chloride) (PVC) of suspension grade No. SG660, with a K value of 66, an apparent bulk density of 0.55 g mL^−1^ and an impurity of less than 5%, was used as the polymer matrix (Thailand Plastic and Chemical Company, Bangkok, Thailand). Several additives were added to the PVC powder to improve the melt processing before compounding. The additives in the formulation of the PVC compound were calcium stearate (632.33 g mol^−1^ molecular weight, Sigma-Aldrich, Taufkirchen, Germany) and zinc stearate (607.02 g mol^−1^ molecular weight, Sigma-Aldrich, Taufkirchen, Germany) as thermal stabilizers and epoxidized soybean oil (bio-based plasticizer, Chemmin Company, Bangkok, Thailand). The physical characteristics of ESBO were 6.5 g mL^−1^ and oxirane functionality 6%. The interfacial agent ϒ-aminopropyltriethoxysilane (ATPS, molecular weight of 221.37 g mol^−1^ and density of 0.9 g mL^−1^ at 25 °C, purity ≥ 98%, Sigma Aldrich, Taufkirchen, Germany) and polypropylene graft maleic anhydride (PP-g-MAH, density of 0.903 g cm^−3^ and MFI of 49 g 10 min^−1^ at 190 °C and 2.16 kg, Fusabond^TM^ P613, Dow chemical Company, Saint Louis, MO, USA) were used in this study. The chemical structures of all interfacial agents are shown in Figure 1.

**Figure 2:**
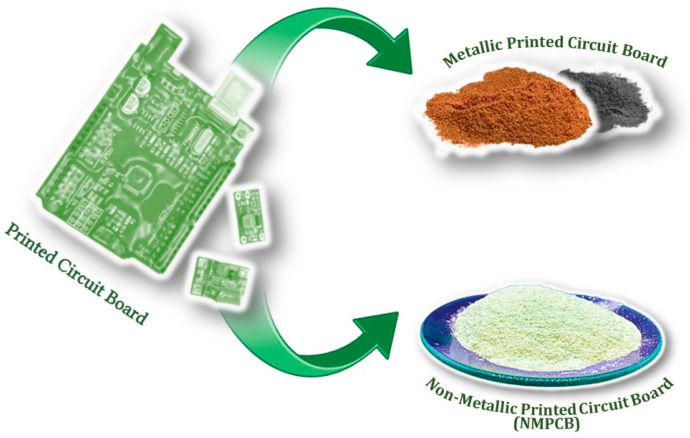
Separation of waste printed circuit board into metallic and non-metallic powders.

In this study, the reused parts and the toxic components were disassembled from the post industry waste PCB before crushing. NMPCB powder (180 µm in size, mesh No. 80 sieve) of post industry waste PCB (FR-4) was selected as the experimental material and obtained from the department of hazardous and waste management, Chulalongkorn University, Thailand, shown in Figure 2.

### 2.2. Surface Treatment of NMPCB Composites Compounding

The surface of the recycled NMPCB powder was modified with 1 wt.% content of the silane interfacial agent (ϒ-aminopropyltriethoxysilane, ATPS) for better interfacial adhesion between the PVC matrix and the surface of the NMPCB by means of silanization for 1 h at 80 °C under agitation and reflux conditions. Prior to the silanization process, the silane interfacial agent solution was developed using 1 wt.% of ATPS (based on the weight of NMPCB) mixed with ethanol solution (ethanol–deionized water, volume ratio 70:30) for 30 min at 500 rpm with a magnetic stirrer. The NMPCB suspension was then filtered. The surface-treated NMPCB powder was further dried in an oven at 70 °C for 24 h before subsequently being used in the PVC/NMPCB composites.

### 2.3. Composites Compounding

All composites were prepared by melt mixing the compatibilizer treated (PP-g-MAH and ATPS) and untreated NMPCB in a HAAKE Rheomic with two roller blades and a 60 cm^3^ mixing chamber of volumetric capacity under the processing condition of 150 °C and 60 RPM for 6 min. Each batch of the composite contained various NMPCB loading from 10, 20 and 30 wt.% and without or with compatibilizer treated 1 wt.% PP-g-MAH or 1 wt.% ATPS. 

### 2.4. Characterization of PVC/NMPCB Composites

The interaction of NMPCB with different interfacial agents (APTS and PP-g-MAH) and functional groups of the PVC in PVC composite was studied using FTIR (Perkin Elmer Spectrum One) equipment with an attenuated total reflectance (ATR) accessory. The spectra were recorded using absorbance mode in the frequency range of 650–4000 cm^−1^, with a resolution of 4.0 cm^−1^. The density of neat PVC and PVC/NMPCB composites was determined through the utilization of Archimedes’ principle by measurement of the weight of the specimen in air and in water at room temperature according to ASTM standard D792 on a Sartorius balance. Distilled water was used as the liquid and at least three samples were measured for each result. Micrographs from SEM analysis were obtained to examine surface morphology of fractured specimens and the NMPCB-PVC matrix interface bonding samples of PVC composites using SEM (JEOL, Hitachi, Model S4800, Tokyo, Japan). Samples were sputter-coated with a thin layer of gold to prevent electronic charge formation during the analysis.

The tensile tests of PVC/NMPCB composites were carried out according to ASTM standard D882. Five replicate samples with dimensions of 120 mm × 10 mm × 0.8 mm (length × width × thickness) were tested using a Lloyd universal testing machine (type LDX, Bognor Regis, West Sussex, UK) with crosshead speed of 50 mm min^−1^ and gauge length of 50 mm. In the study of dynamic mechanical analysis, the samples with dimensions of 50 mm × 3 mm × 3 mm (length × width × thickness) were performed using a DMA (GABO, EplexorR, type 100 N, Ahlden, Germany) with the tensile mode of the equipment. The viscoelastic properties of the samples were determined under the conditions of the temperature ranging between 10 °C and 140 °C, with a heating rate of 1 °C min^−1^, under nitrogen flow and at a fixed frequency of 1 Hz of static strain of 0.2%.

## 3. Results and Discussion

PVC/NMPCB composites were successfully prepared by varying the amount of NMPCB between 10, 20 and 30 wt.% by using an internal mixer. For better interfacial adhesion between the PVC matrix and the surface of the NMPCB, polypropylene grafted maleic anhydride (PP-g-MAH) and ϒ-aminopropyltriethoxy silane (ATPS) were used as an interfacial agent. 

### 3.1. FTIR of Untreated and Treated PVC/NMPCB Composites

**Figure 3:**
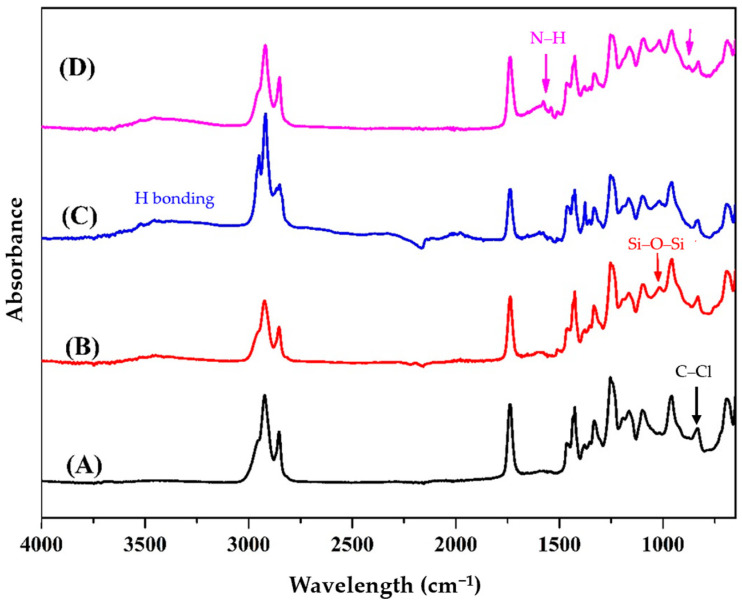
FTIR-ATR spectra of (**A**) neat PVC, (**B**) untreated PVC/NMPCB composites, (**C**) PP-g-MAH treated PVC/NMPCB composites and (**D**) ATPS treated PVC/NMPCB composites.

The FTIR-ATR spectra of PVC, untreated PVC/NMPCB composite and PVC/NMPCB composites treated with PP-g-MAH and ATPS are shown in Figure 3. The FTIR-ATR spectrum of neat PVC shows absorption peaks at 2923 and 2853 cm^−1^, corresponded to C–H stretching vibration of CH_2_; 1738 cm^−1^ corresponded to the C=O from bio-based plasticizer—ESBO; 1332 cm^−1^, which can be assigned to C–H deformation of CH_2_; 1254 cm^−1^ of C–H rocking; and 833 cm^−1^ of C–Cl, which was reported in the study by V.V. Soman et al. [26]. These are the characteristic peaks of neat PVC. The untreated PVC/NMPCB composite showed the appearance of a new absorption peak at wave number 1016 cm^−1^, which is the characteristic peak of Si–O–Si in NMPCB, indicating the presence of NMPCB powder in the PVC matrix. The slight shift of carbonyl peak and C–Cl peak showed no significant change in the functional groups of untreated PVC composite when compared with neat PVC. This result indicated poor interaction between the NMPCB powder and the PVC matrix, which was in agreement with the literature [24]. In Figure 3C, PVC composite with PP-g-MAH shows the appearance of a new absorption peak at 3208 cm^−1^, representing the formation of hydrogen bonding between the PVC and the maleic anhydride group of PP-g-MAH. There are strong peaks between 2926 and 2855 cm^−1^, which related to the C–H stretching vibration of CH_2_ and CH_3_ groups due to the aliphatic carbons of the PP-g-MAH compatibilizer [27,28,29], implying that the surface of NMPCB was modified using the interfacial agent. In Figure 3D, the appearance of small peaks at 1577 cm^−1^ and 1379 cm^−1^ corresponded to the bending vibration of N–H from ATPS on the NMPCB surface and the peak at 872 cm^−1^ represents the –Si–NMPCB bond, which is the evidence of surface modification of the NMPCB using ATPS. Another low intensity peak was found at 781 cm^−1^, which corresponded to the –Si–O bond [30,31,32,33,34,35]. The peak at 872 cm^−1^ representing –Si–NMPCB bond, was consistence with a report which revealed the bonding between –Si–polar functional group on fibers [36]. These findings suggested successful functionalization of NMPCB (surface modification of NMPCB) was occurred due to ATPS. Similar results were also observed in the literature in which the interaction between C–Cl of PVC and the amino functional group of ATPS modified zeolites resulted in the slight shifting of the C–Cl peak to a lower wavenumber [37]. These results confirmed the interaction between the PVC molecular chain and the ATPS surface treated NMPCB. The FTIR absorption peaks at 1057 and 783 cm^−1^ indicated the presence of a highly condensed silica-containing asymmetric and symmetric siloxane (Si–O–Si) network mixture.

### 3.2. Density of Untreated and Treated PVC/NMPCB Composites

**Figure 4:**
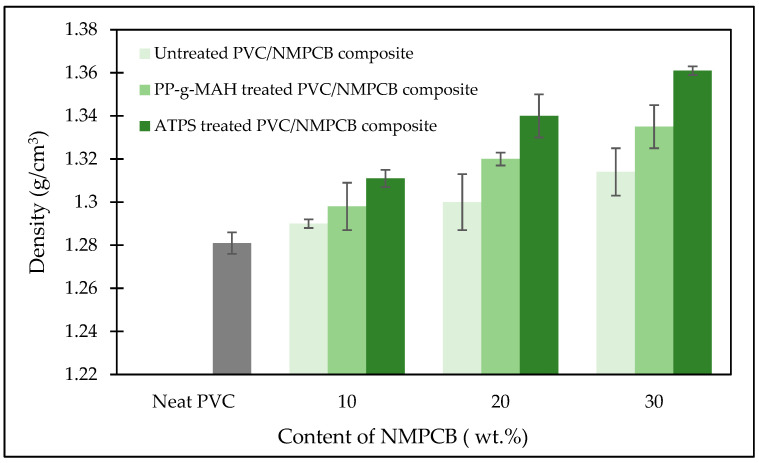
Effect of different interfacial agents on density of PVC/NMPCB composites.

Figure 4 shows the density of PVC/NMPCB composites with different contents of NMPCB and interfacial agents. Density was found to increase with the addition of NMPCB powder. This is due to the higher amount of NMPCB, which was corresponding to the higher density of NMPCB and caused the increase in the density of untreated PVC/NMPCB composites. NMPCB powder showed rigid characteristics and was heavier than PVC resin. The presence of PP-g-MAH in PVC/NMPCB composites promoted the interfacial adhesion. This was shown by the slightly greater increase in density. On the other hand, the PVC/NMPCB composites treated with ATPS resulted in a significant increase in density when compared with the untreated PVC/NMPCB composites and the PVC/NMPCB composites treated with PP-g-MAH. This considerable increment of the density was justified by the enhancement of interfacial adhesion between the NMPCB powder and PVC matrix, resulting in the decrease in void or gap formation and was probably due to the better dispersion and interfacial adhesion in the PVC matrix. These results were consistent with the SEM morphology analysis. It was inferred that ATPS could enhance the better mechanical properties of the PVC/NMPCB composites.

### 3.3. Morphology Investigation for Interfacial Adhesion on Untreated and Treated PVC/NMPCB Composites

**Figure 5:**
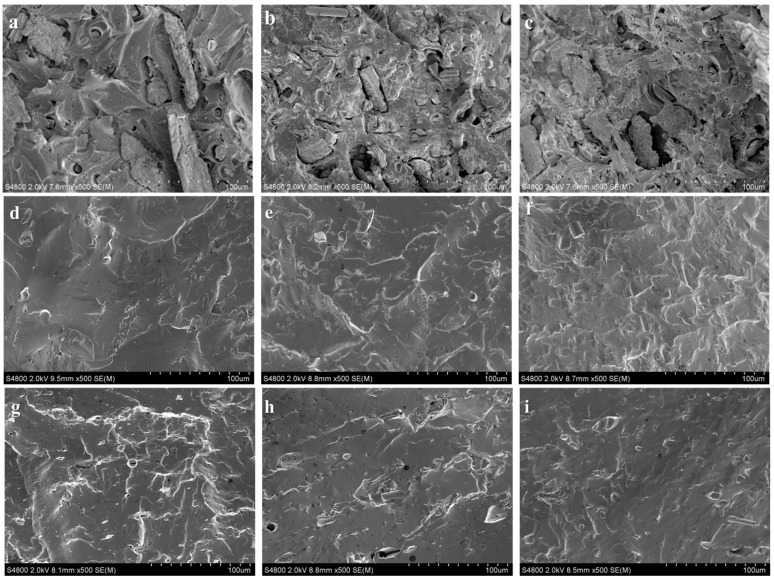
SEM images of PVC/NMPCB composites (**a**) untreated 10 wt.% NMPCB; (**b**) untreated 20 wt.% NMPCB; (**c**) untreated 30 wt.% NMPCB; (**d**) 10 wt.% NMPCB treated ATPS; (**e**) 20 wt.% NMPCB treated ATPS; (**f**) 30 wt.% NMPCB treated ATPS; (**g**) 10 wt.% NMPCB treated PP-g-MAH, (**h**) 20 wt.% NMPCB treated PP-g-MAH and (**i**) 30 wt.% NMPCB treated PP-g-MAH.

SEM micrographs of the surface of PVC/NMPCB composites with different interfacial agents are shown in Figure 5. In general, the morphology of a material can be used to identify information about its mechanical properties and interactions between the disperse and matrix phase i.e., NMPCB and PVC. The NMPCB/PVC composite without an interfacial agent showed glass fiber surfaces drawn with voids due to weaker interfacial adhesion between the PVC matrix and NMPCB filler, which resulted in poor mechanical properties and characteristics. In the untreated PVC/NMPCB composites, more voids formed around the NMPCB and holes left by glass fiber pullout in the matrix were observed at higher NMPC contents. This result was consistent with the low interfacial adhesion between the polymer matrix and the NMPCB filler because of the poor chemical interaction or compatibility. Furthermore, the increasing contents of NMPCB made for poor flow ability and dispersion, resulting in the poor interfacial adhesion [38]. In the presence of ATPS in PVC/NMPCB composites, the void formation and holes were seen to be more heterogeneous than those in the untreated PVC/NMPCB composites, as shown in Figure 5d–f. The polymer matrix was shown to significantly increase in terms of the amount of embedded glass fibers. These observations provided qualitative evidence for the improvement of interfacial adhesion after surface modification of NMPCB using ATPS., thus, being indicative of the improved tensile strength and modulus as compared with untreated PVC/NMPCB composites. On the other hand, the NMPCB dispersion and interfacial adhesion were improved by the incorporation of PP-g-MAH in PVC/NMPCB composites when compared with the untreated PVC/NMPCB composites (see Figure 5g–i). Hence, the better interfacial adhesion between NMPCB and PVC matrixes was achieved when the PVC composites were treated with PP-g-MAH and ATPS. However, the difference in the NMPCB surface wettability or the interfacial adhesion capability of NMPCB was most likely attributed to the difference in the reactivity of functional groups within the two interfacial agents, which play a vital role in the improvement of mechanical properties of PVC/NMPCB composites.

### 3.4. Mechanical Properties of the Untreated and Treated PVC/NMPCB Composites

**Figure 6:**
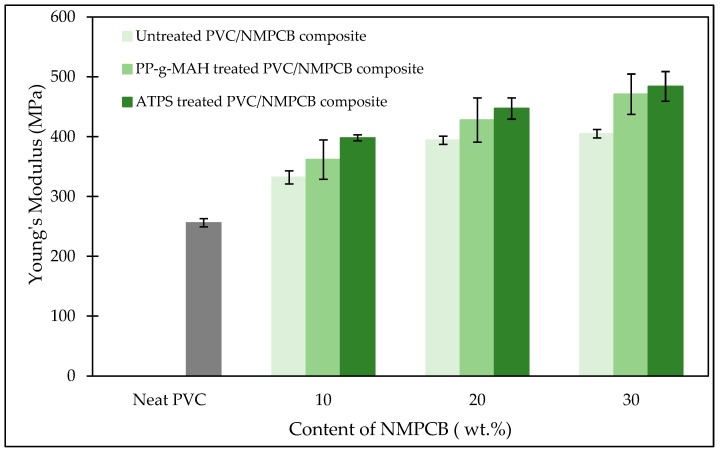
Young’s modulus of untreated and treated PVC/NMPCB composites.

**Figure 7:**
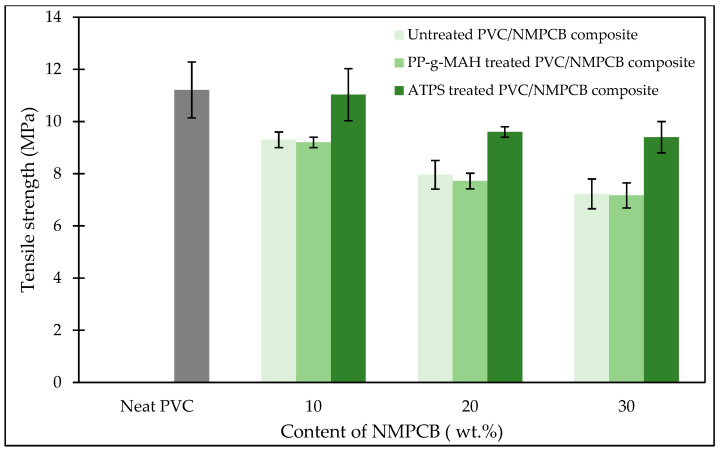
Tensile strength of untreated and treated PVC/NMPCB composites.

**Figure 8:**
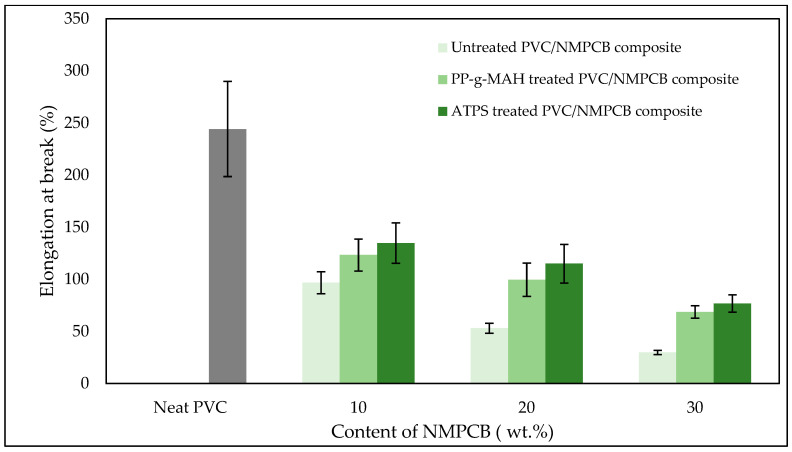
Elongation at break of untreated and treated PVC/NMPCB composites.

Mechanical properties of untreated and treated PVC/NMPCB composites were investigated using a tensile test to determine the effect of the incorporation of NMPCB in the range of 0 to 30 wt.% and under treatment with different interfacial agents, on Young’s modulus, tensile strength and elongation at break (see Figure 6, Figure 7 and Figure 8). In Figure 6, Young’s modulus was 256 MPa for neat PVC and 332 MPa, 394 MPa and 405 MPa for the untreated PVC/NMPCB composites with 10, 20 and 30 wt.% of NMPCB contents, respectively. This could be attributed to the rigid nature of NMPCB, which could promote improve stiffness for the composite. The addition of NMPCB in PVC matrixes might disturb the mobility of PVC molecular chains and consequently produce a more rigid composite which is consistent with the previous studies [21,24,39]. When the NMPCB was treated with interfacial agents, improvement in Young’s modulus was observed in this work. However, the more extensive improvement in Young’s modulus was recorded in PVC/NMPCB composites treated with ATPS from 10, 20 and 30 wt.% with the increment of 19.87%, 13.45% and 19.50% as compared with untreated PVC/NMPCB composites. For PP-g-MAH treated with PVC/NMPCB composites, it showed an increment in Young’s modulus of between 8.98%, 8.55% and 16.29%. These results could be explained by the fact that the improvement in stiffness (Young’s modulus) occurred when the fillers were better dispersed [40].

Figure 7 shows the effect of the content of NMPCB and interfacial agents on the tensile strength of PVC/NMPCB composites. The larger decrease in tensile strength was observed when more NMPCB content was added to PVC/NMPCB composites. This was probably due to the hinderance on PVC molecular chain mobility produced by the increase in the content of glass fiber from NMPCB, resulting in the poor dispersion of NMPCB in PVC matrixes. This result could also be explained by the irregular shape of filler resulting in poor stress transfer ability in the polymer matrix [41]. Consequently, the decline in tensile strength in the PVC composites with the varying content of NMPCB from 10, 20 and 30 wt.% resulted from the poor stress transfer which was consistence with the previous studies [10,38,42]. It is worth noting that the tensile strength increased from 9.3 MPa for 10 wt.% of untreated PVC/NMPCB composite to 11.3 MPa for the PVC/NMPCB composites with an ATPS interfacial agent, which was close to the tensile strength of the neat PVC, representing a 21.50% increment. Furthermore, when ATPS was employed as an interfacial agent the tensile strength of the PVC/NMPCB composite increased from 7.96 MPa (without interfacial agent) to 9.60 MPa (at 20 wt.% NMPCB) and from 7.23 MPa (without interfacial agent) to 9.40 MPa (at 30 wt.% NMPCB). When PP-g-MAH was employed as an interfacial agent for PVC/NMPCB composites, the tensile strength of the PVC/NMPCB composites was relatively similar to that of the composite without interfacial agent. This phenomenon was likely due to the poor compatibility of the PP-g-MAH interfacial agent because of the different polarity between the nonpolar nature of polypropylene from PP-g-MAH and the polar polymer of PVC chain. Therefore, it could be observed that the efficiency of the interfacial agents for PVC/NMPCB composites was different, depending on the nature of the interfacial agents.

Figure 8 shows the effect of the contents of NMPCB and interfacial agents on the elongation at break of PVC/NMPCB composites. From the figure, neat PVC showed ductile failure with the elongation at break of 250%. The higher contents of NMPCB in untreated PVC/NMPCB composites showed the larger decrease in elongation at break when compared with neat PVC. This result could be attributed to the lower elasticity properties of NMPCB, which caused the reduce mobility of the PVC molecular chain impeding large movements by tensile stress, and resulting in inducing the sample specimens to break at a lower value of deformation [40]. When PVC/NMPCB composites were treated with interfacial agent, the elongation at break became higher than the untreated PVC/NMPCB composites. This result could probably be due to the better dispersion of NMPCB and improved interfacial adhesion. However, the PVC/NMPCB composites treated with ATPS showed the highest elongation at break. Such improvement of elongation at break was related to the stronger interfacial adhesion between the NMPCB and PVC matrix as shown in SEM micrographs (Figure 5), consequently a better optimized transfer of stresses from the PVC matrix to the NMPCB filler occurred.

### 3.5. Dynamic Mechanical Properties of Untreated and Treated PVC/NMPCB Composites

**Figure 9:**
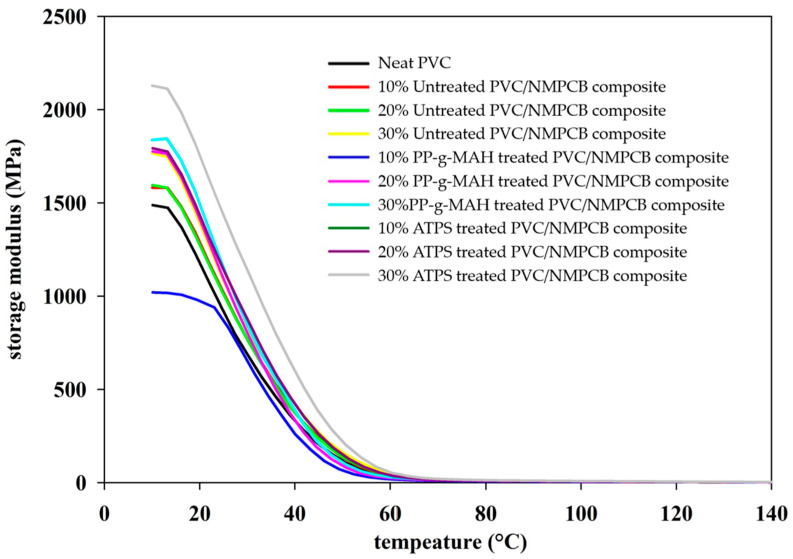
Effect of interfacial agents on storage modulus of PVC/NMPCB composites.

**Figure 10:**
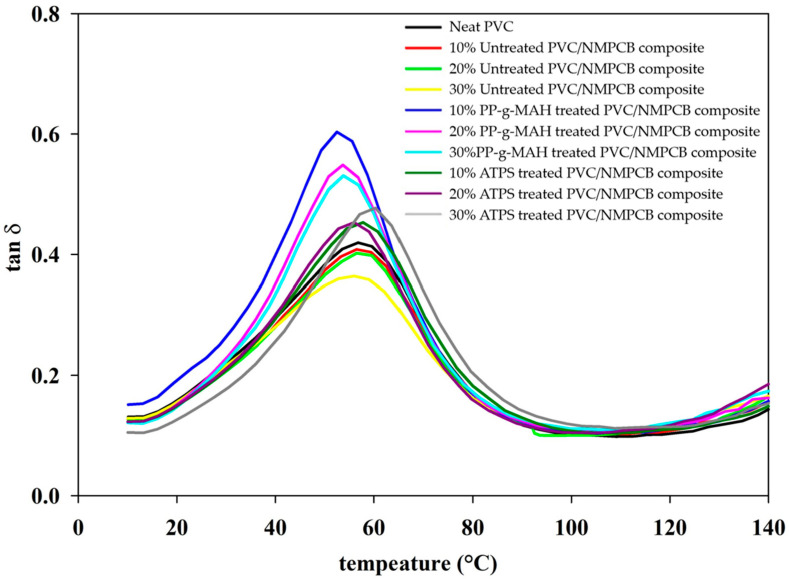
Effect of interfacial agents on the loss factor or tan δ of PVC/NMPCB composites.

To understand the effect of NMPCB content and the interfacial adhesion property (compatibility) of interfacial agents on PVC matrixes, the dynamic mechanical properties of PVC/NMPCB composites are presented in Figure 9 and Figure 10. The result of the storage modulus from untreated PVC/NMPCB composites provided the information on rigidity or stiffness of the material, which was consistent with the previous studies [10,42]. The storage modulus increased with an increase in NMPCB content. Consequently, the storage modulus of 30 wt.% NMPCB in PVC/NMPCB composites increased by up to 43.04% compared with neat PVC in the glassy state as shown in Figure 9. An increase in the storage modulus was observed up to 60 °C in the rubbery region. This result was associated with low molecular chain mobility due to the incorporation of NMPCB. The effect of the incorporation of NMPCB in PVC/NMPCB composites on the storage modulus revealed insignificant change with increasing temperature. This observation was likely due to be the weakening of the interfacial adhesion, formation of higher movement of molecular chain in free volume [43,44] and the effect of plasticizer under thermal energy [10].

It was also observed that the storage modulus of PP-g-MAH treated PVC/NMPCB composites with 10 wt.% NMPCB was less than that of neat PVC in the glassy region, as shown in Figure 9. This result was likely due to the effect of the flexibility of PP-g-MAH that was covered with NMPCB. A similar result was also observed in the literature [45], which suggested that the lower stiffness value in the maleic acid grafted polymer composite was because of the formation of a stiffer polymer phase around the filler or fiber without interfacial agent or in the changed fiber orientation caused by interfacial agent. V. Hristov et al. reported that PP-g-MAH had the characteristics of a thin, plastically interfacial layer formation and had deformation properties. With an increase in temperature, it became softer and more ductile than the bulk matrix, resulting in lower storage modulus value [46]. With the incorporation of NMPCB content of 20 wt.% and 30 wt.%, the storage modulus reached a higher value when compared to the same content of NMPCB in PVC/NMPCB composites, which indicated an enhanced stiffness. This result implied that the interaction between the NMPCB and PVC matrix in the presence of PP-g-MAH became obvious with the increasing content of NMPCB owing to the high possibility of physical contact. It could be suggested that the plasticizing and dispersion capability of the PP-g-MAH signified in the incorporation of 10 wt.% NMPCB in PVC/NMPCB composites (the plasticizing and dispersion capability of PP-g-MAH was affected by the content of NMPCB).

For ATPS treated PVC/NMPCB composites, it was clearly observed that the storage modulus improved when compared with untreated PVC/NMPCB composites and neat PVC in the glassy region because the interfacial adhesion was significantly imparted on the storage module at a temperature (in glassy region) lower than the glass transition temperature due to freezing and warping between the polymer matrix chain and filler. The storage modulus of ATPS treated PVC/NMPCB composites increased in the glassy as well as the rubbery region. This result was associated with the improved interfacial adhesion between the NMPCB and the PVC, resulting in better stress transfer between the NMPCB and the PVC molecular chain. However, the occurrence of the mobility and the deformation of the polymer chain increased with an increase in the temperature under the action of external forces. Consequently, the weakening of the interfacial adhesion caused the diminishing of the storage modulus.

When comparison interfacial agents, it can be seen that the storage modulus of PVC/NMPCB composites in the presence of ATPS were higher when PP-g-MAH was employed, which showed that ATPS improved the interfacial adhesion between the NMPCB and the PVC matrix. These results are in agreement with the micrographs taken in the SEM analysis and reported in Figure 5.

The damping factor or loss factor of the composites was obtained from the ratio of loss modulus (E″) to the storage modulus (E′). For PVC/NMPCB composites, the study of tan δ is required to understand the fiber or filler-polymer matrix adhesion level. The peak of loss factor or tan δ represented the glass transition temperature at which material starts softening. Under the temperature range from 0 °C to 140 °C, a single glass transition was observed in all the neat PVC, untreated PVC/NMPCB composites and treated PVC/NMPCB composites with different interfacial agents, as shown in Figure 10. Generally, the incorporation of reinforcing filler decreases the damping factor. It could be observed that the loss factor or damping factor (tanδ) was inversely proportional to the NMPCB contents. Therefore, the magnitude of loss factor or tan δ of 30 wt.% untreated PVC/NMPCB composites was lower than neat PVC since the incorporation of NMPCB restricted the movement of the PVC molecular chain. This result was consistent with the dynamic mechanical analysis of polymer composites from previous studies [10,47]. There was no change in the tan δ_max_ peak value (T_g_) in untreated PVC/NMPCB composites.

The comparison of PVC/NMPCB composites with different interfacial agents showed that the peaks of the tan δ of PVC/NMPCB composites with PP-g-MAH, interpreted as the glass transition temperature (T_g_), shifted slightly to a lower temperature position when compared with that of neat PVC. This shifting might be associated with the chain flexibility of PP-g-MAH interacting with hydroxyl of bisphenol in the epoxy resin or Si-OH of glass fiber from NMPCB (type FR-4), which resulted in the formation of hydrogen bonding between the NMPCB and PP-g-MAH. It was seen that the higher contents of NMPCB in PVC/NMPCB composites hindered the movement of the PVC molecular chain with a decrease in the peak intensity. The glass transition temperature of PP-g-MAH was completely absent in the PVC/NMPCB composites. This result indicated that PP-g-MAH was completely miscible in the PVC matrix.

It was well known that the PVC could interact with the amino group of ATPS via an acid-base interaction [48]. Several studies found that the amino functional group of silanes interfacial agent treated fillers were effective interfacial agents for well-dispersed fillers in the PVC matrix and improved interfacial adhesion between the fillers and the PVC molecular chain in PVC/NMPCB composites [49,50,51]. Consequently, ATPS was employed in the study to treat the NMPCB surface. When NMPCB was treated with ATPS, tan δ peak shifted to a higher temperature position (60 °C) compared with that of neat PVC, untreated PVC/NMPCB composites and PP-g-MAH treated PVC/NMPCB composites. It was clearly observed that the curve of loss factor moved to a higher temperature region. This resulted from the restriction of the PVC chain brought about to some extent by the stronger interfacial adhesion between the NMPCB and PVC molecular chain, resulting from the interaction between the amino groups of ATPS treated NMPCB, which acted as the electron donor and carbon cations, in the PVC molecular chain after losing a Cl^−^ ion due to heating, which acted as the electron acceptor. G. Chen et al. studied the interfacial adhesion of PVC/ATPS treated SiO_2_ composite using dynamic mechanical analysis. The increased in the peak loss factor or tan δ with increasing T_g_ was observed. This explained the stronger interfacial adhesion and the larger volume fraction of filler particle [52]. A. Zhu et al. found that the shifting of tan δ peak to the higher temperature position corresponded with the enhanced interfacial adhesion between PMMA treated SiO_2_ and PVC matrixes [50].

According to dynamic mechanical analysis, the shifting of loss factor represented the improved interfacial adhesion between the NMPCB and PVC matrix as compared with that of untreated PVC/NMPCB composites. These results could be interpreted as the effect of different interfacial agents on the dynamic mechanical analysis of PVC/NMPCB composites, the stronger interfacial adhesion between the NMPCB and PVC matrix being associated with the shifting of T_g_ to a higher temperature position within the higher temperature region.

It was worth noting that the intensity and value of the tan δ_max_ peak were affected by the content of NMPCB and the addition of compatibilizers in the PVC/NMPCB composites as shown in Figure 10. The addition of ATPS gave the higher increase in T_g_ with the lower tan δ and broader peak, which indicated that the PVC/NMPCB composites gave stronger interfacial adhesion than the PVC/NMPCB composites incorporated with PP-g-MAH, resulting from greater restrictions in the PVC molecular chain motion beyond the polymer–NMPCB interface. However, the addition of PP-g-MAH gave the plasticizing characteristic with the shifting of T_g_ to the lower temperature range due to the flexibility interface.

## 4. Conclusions

This research utilized different interfacial agents, with different chemical structures, commonly used in industry and relatively inexpensive. Different methods of surface modification and the effect of the incorporation of NMPCB in PVC on interfacial adhesion and mechanical properties were investigated. The interaction between ATPS treated NMPCB with PVC matrixes and PP-g-MAH treated NMPCB with PVC matrixes was studied using FTIR-ATR analysis. The density of PVC/NMPCB composites increased with the additional of an amount of treated NMPCB with both ATPS and PP-g-MAH, of up to 1.34 and 1.36 g cm^−3^ at 30 wt.% NMPCB treated with ATPS and PP-g-MAH, respectively. The SEM morphology of the PVC/NMPCB treated ATPS composites and PVC/NMPCB treated PP-g-MAH composites showed good distribution and dispersion of the NMPCB powders in the PVC matrix. The mechanical properties, Young’s modulus, tensile strength and elongation at break, of PVC/NMPCB composites increased with the addition of NMPCB treated ATPS. The maximum Young’s modulus of PVC/NMPCB composites was achieved at 30 wt.% ATPS treated NMPCB at 484 MPa. The dynamic mechanical analysis also confirmed these observations, showing an increase in the storage modulus in the PVC/NMPCB composite with the incorporation of 30 wt.% of both the PP-g-MAH and ATPS treated NMPCB. The T_g_ of ATPS treated PVC/NMPCB composite was higher than that of untreated and PP-g-MAH treated composite. This result indicated the strong interfacial adhesion between the ATPS treated NMPCB filler and the PVC matrix. It could be concluded that the ATPS interfacial agent showed higher efficiency in terms of the dispersion and interaction between the NMPCB filler and the PVC matrix than PP-g-MAH, and this method of surface modification could easily be adopted in the industry.

## Data Availability

The authors confirm that the data supporting in this study are available within the article. Raw data that support the findings of this study are available from the corresponding author, upon reasonable request.

## References

[1] Li J., Lopez B.N., Liu L., Zhao N., Yu K., Zheng L. (2013). Regional or global WEEE recycling. Where to go?. Waste Manag..

[2] De Marco I., Caballero B., Chomón M., Laresgoiti M., Torres A., Fernández G., Arnaiz S. (2008). Pyrolysis of electrical and electronic wastes. J. Anal. Appl. Pyrolysis.

[3] Guo J., Guo J., Xu Z. (2009). Recycling of non-metallic fractions from waste printed circuit boards: A review. J. Hazard. Mater..

[4] Quan C., Li A., Gao N. (2012). Research on pyrolysis of PCB waste with TG-FTIR and Py-GC/MS. J. Therm. Anal. Calorim..

[5] Yang J., Wang H., Zhang G., Bai X., Zhao X., He Y. (2019). Recycling organics from non-metallic fraction of waste printed circuit boards by a novel conical surface triboelectric separator. Resour. Conserv. Recycl..

[6] Quan C., Li A., Gao N., Dan Z. (2010). Characterization of products recycling from PCB waste pyrolysis. J. Anal. Appl. Pyrolysis.

[7] Alenezi R.A., Al-Fadhli F.M. (2018). Thermal degradation kinetics of waste printed circuit boards. Chem. Eng. Res. Des..

[8] Xiu F.R., Zhang F.S. (2010). Materials recovery from waste printed circuit boards by supercritical methanol. J. Hazard. Mater..

[9] Yildirir E., Onwudili J.A., Williams P.T. (2015). Chemical Recycling of Printed Circuit Board Waste by Depolymerization in Sub- and Supercritical Solvents. Waste Biomass Valorization.

[10] Moe A.K., Chungprempree J., Preechawong J., Sapsrithong P., Nithitanakul M. (2022). Recycling Waste Nonmetallic Printed Circuit Boards for Polyvinyl Chloride Composites. Polymers.

[11] Xiu F.-R., Weng H., Qi Y., Yu G., Zhang Z., Zhang F.-S. (2016). A novel reutilization method for waste printed circuit boards as flame retardant and smoke suppressant for poly (vinyl chloride). J. Hazard. Mater..

[12] Senophiyah-Mary J., Loganath R. (2020). A novel method of utilizing waste Printed Circuit Board for the preparation of Fibre Reinforced Polymer. J. Clean. Prod..

[13] Rajagopal R.R., Aravinda L.S., Rajarao R., Bhat B.R., Sahajwalla V. (2016). Activated carbon derived from non-metallic printed circuit board waste for supercapacitor application. Electrochim. Acta.

[14] Yang S., Bai S., Wang Q. (2015). Preparation of fine fiberglass-resin powders from waste printed circuit boards by different milling methods for reinforcing polypropylene composites. J. Appl. Polym. Sci..

[15] Hu D., Jia Z., Li J., Zhong B., Fu W., Luo Y., Jia D. (2018). Characterization of Waste Printed Circuit Boards Nonmetals and its Reutilization as Reinforcing Filler in Unsaturated Polyester Resin. J. Polym. Environ..

[16] Shin S.-R., Mai V.D., Lee D.-S. (2019). Chemical Recycling of Used Printed Circuit Board Scraps: Recovery and Utilization of Organic Products. Processes.

[17] Xian J., Li M., Lin Z., Deng S. (2017). Crystallization and thermal behavior of recycled polypropylene composites containing nonmetallic printed circuit board powder and β-nucleating agents. J. Therm. Anal. Calorim..

[18] Biswal M., Jada N., Mohanty S., Nayak S.K. (2015). Recovery and utilisation of non-metallic fraction from waste printed circuit boards in polypropylene composites. Plast. Rubber Compos..

[19] Barczewski M., Matykiewicz D., Andrzejewski J., Skórczewska K. (2016). Application of waste bulk moulded composite (BMC) as a filler for isotactic polypropylene composites. J. Adv. Res..

[20] Grigorescu R.M., Ghioca P., Iancu L., David M.E., Andrei E.R., Filipescu M.I., Ion R.-M., Vuluga Z., Anghel I., Sofran I.-E. (2020). Development of thermoplastic composites based on recycled polypropylene and waste printed circuit boards. Waste Manag..

[21] Yang S., Bai S., Wang Q. (2016). Morphology, mechanical and thermal oxidative aging properties of HDPE composites reinforced by nonmetals recycled from waste printed circuit boards. Waste Manag..

[22] Sun Z., Shen Z., Zhang X., Ma S. (2015). Co-recycling of acrylonitrile–butadiene–styrene waste plastic and nonmetal particles from waste printed circuit boards to manufacture reproduction composites. Environ. Technol..

[23] Grigorescu R.M., Ghioca P., Iancu L., David M.E., Ion R.-M., Nicolae C.-A., Gabor R.A., Radu E.R., Ganciarov M., Spurcaciu B. (2022). Influence of non-metallic fraction of printed circuit boards waste on recycled polyvinyl chloride from waste wires. J. Appl. Polym. Sci..

[24] Das R.K., Gohatre O.K., Biswal M., Mohanty S., Nayak S.K. (2019). Influence of non-metallic parts of waste printed circuit boards on the properties of plasticised polyvinyl chloride recycled from the waste wire. Waste Manag. Res..

[25] Wang X., Guo Y., Liu J., Qiao Q., Liang J. (2010). PVC-based composite material containing recycled non-metallic printed circuit board (PCB) powders. J. Environ. Manag..

[26] Soman V.V., Kelkar D.S. (2009). FTIR studies of doped PMMA-PVC blend system. Macromolecular Symposia.

[27] Li Q., Matuana L.M. (2003). Surface of cellulosic materials modified with functionalized polyethylene coupling agents. J. Appl. Polym. Sci..

[28] Lu J.Z., Negulescu I.I., Wu Q. (2005). Maleated wood-fiber/high-density-polyethylene composites: Coupling mechanisms and interfacial characterization. Compos. Interfaces.

[29] Luo G., Li W., Liang W., Liu G., Ma Y., Niu Y., Li G. (2017). Coupling effects of glass fiber treatment and matrix modification on the interfacial microstructures and the enhanced mechanical properties of glass fiber/polypropylene composites. Compos. Part B Eng..

[30] Abdel-Gawad N.M., El Dein A.Z., Mansour D.-E.A., Ahmed H.M., Darwish M., Lehtonen M. (2018). Multiple enhancement of PVC cable insulation using functionalized SiO_2_ nanoparticles based nanocomposites. Electr. Power Syst. Res..

[31] Park S.-J., Jin J.-S., Lee J.-R. (2000). Influence of silane coupling agents on the surface energetics of glass fibers and mechanical interfacial properties of glass fiber-reinforced composites. J. Adhes. Sci. Technol..

[32] Asyraf M., Rafidah M., Azrina A., Razman M. (2021). Dynamic mechanical behaviour of kenaf cellulosic fibre biocomposites: A comprehensive review on chemical treatments. Cellulose.

[33] Herrera-Franco P., Valadez-Gonzalez A. (2005). A study of the mechanical properties of short natural-fiber reinforced composites. Compos. Part B Eng..

[34] Demir H., Atikler U., Balköse D., Tıhmınlıoğlu F. (2006). The effect of fiber surface treatments on the tensile and water sorption properties of polypropylene–luffa fiber composites. Compos. Part A Appl. Sci. Manuf..

[35] Kucuk F., Sismanoglu S., Kanbur Y., Tayfun U. (2020). Effect of silane-modification of diatomite on its composites with thermoplastic polyurethane. Mater. Chem. Phys..

[36] Yu T., Ren J., Li S., Yuan H., Li Y. (2010). Effect of fiber surface-treatments on the properties of poly (lactic acid)/ramie composites. Compos. Part A Appl. Sci. Manuf..

[37] Jamshaid F., Dilshad M.R., Islam A., Khan R.U., Ahmad A., Adrees M., Haider B. (2020). Synthesis, characterization and desalination study of polyvinyl chloride-co-vinyl acetate/cellulose acetate membranes integrated with surface modified zeolites. Microporous Mesoporous Mater..

[38] Guo J., Tang Y., Xu Z. (2010). Performance and thermal behavior of wood plastic composite produced by nonmetals of pulverized waste printed circuit boards. J. Hazard. Mater..

[39] Li S., Sun S., Liang H., Zhong S., Yang F. (2014). Production and characterization of polypropylene composites filled with glass fibre recycled from pyrolysed waste printed circuit boards. Environ. Technol..

[40] Franco-Marquès E., Méndez J., Pèlach M., Vilaseca F., Bayer J., Mutjé P. (2011). Influence of coupling agents in the preparation of polypropylene composites reinforced with recycled fibers. Chem. Eng. J..

[41] Azizah A., Rozman H., Azniwati A., Tay G. (2020). The effect of filler loading and silane treatment on kenaf core reinforced polyurethane composites: Mechanical and thermal properties. J. Polym. Environ..

[42] Muniyandi S.K., Sohaili J., Hassan A. (2016). Accelerated weathering properties of compatibilized composites made from recycled HDPE and nonmetallic printed circuit board waste. J. Appl. Polym. Sci..

[43] Landel R.F., Nielsen L.E. (1993). Mechanical Properties of Polymers and Composites.

[44] Sun S., Li C., Zhang L., Du H., Burnell-Gray J. (2006). Effects of surface modification of fumed silica on interfacial structures and mechanical properties of poly (vinyl chloride) composites. Eur. Polym. J..

[45] Wielage B., Lampke T., Utschick H., Soergel F. (2003). Processing of natural-fibre reinforced polymers and the resulting dynamic–mechanical properties. J. Mater. Process. Technol..

[46] Hristov V., Vasileva S. (2003). Dynamic mechanical and thermal properties of modified poly (propylene) wood fiber composites. Macromol. Mater. Eng..

[47] Tajvidi M., Falk R.H., Hermanson J.C. (2006). Effect of natural fibers on thermal and mechanical properties of natural fiber polypropylene composites studied by dynamic mechanical analysis. J. Appl. Polym. Sci..

[48] Matuana L.M., Woodhams R.T., Balatinecz J.J., Park C.B. (1998). Influence of interfacial interactions on the properties of PVC/cellulosic fiber composites. Polym. Compos..

[49] Yim H., Kim D.S. (2012). Physical properties of PVC/aminosilane-treated wood flour/organoclay composites. Polym. Adv. Technol..

[50] Zhao Y., Wang K., Zhu F., Xue P., Jia M. (2006). Properties of poly (vinyl chloride)/wood flour/montmorillonite composites: Effects of coupling agents and layered silicate. Polym. Degrad. Stab..

[51] Kokta B., Maldas D., Daneault C., Béland P. (1990). Composites of polyvinyl chloride–wood fibers. III: Effect of silane as coupling agent. J. Vinyl Technol..

[52] Chen G., Tian M., Guo S. (2006). A Study on the Morphology and Mechanical Properties of PVC/nano-SiO2 Composites. J. Macromol. Sci. Part B.

